# Mortality Across Adulthood: The Impact of Cognitive Change

**DOI:** 10.1093/geroni/igaf122.3815

**Published:** 2025-12-31

**Authors:** Olivia Chervenick, Meredith Willard, Nicholas Turiano

**Affiliations:** West Virginia University, Morgantown, West Virginia, United States; West Virginia University, Morgantown, West Virginia, United States; West Virginia University, Morgantown, West Virginia, United States

## Abstract

Cognitive performance is a key indicator of health and may also serve as a predictor of mortality. While previous research has linked lower cognition to increased mortality, less is known about whether changes in certain cognitive abilities are stronger predictors of mortality, and the specific ages in adulthood when changes may be most detrimental. Data from 4,152 individuals from the Midlife Development in the U.S. Study (MIDUS) was utilized. Objective cognitive screeners for episodic memory and executive functioning were obtained at two time points (2005 through 2015) through the Brief Test of Adult Cognition via telephone (BTACT). Utilizing Cox proportional hazards modeling (497 deceased over 7 years), we found that those scoring higher in episodic memory (HR = 0.88) and executive functioning (0.85) were at a significantly decreased risk of dying (all p’s less than .05), controlling for age, gender, race, education level, and marital status. Moreover, those who decreased in episodic memory (HR = 1.50) and executive functioning (HR = 1.14) had a significantly elevated risk of dying. Age moderation analyses were not significant, suggesting that declines in these cognitive abilities universally predicted an increased risk of death for younger and older adults. Overall, reductions in both episodic memory and executive functioning were linked to greater mortality risk across adulthood, regardless of age. Monitoring cognitive change over time could aid in identifying individuals with higher mortality risk, in hopes to create interventions to potentially increase longevity.

